# Analysing Physical Performance Indicators Measured with Electronic Performance Tracking Systems in Men’s Beach Volleyball Formative Stages

**DOI:** 10.3390/s24237524

**Published:** 2024-11-25

**Authors:** Joaquín Martín Marzano-Felisatti, Rafael Martínez-Gallego, José Pino-Ortega, Antonio García-de-Alcaraz, Jose Ignacio Priego-Quesada, José Francisco Guzmán Luján

**Affiliations:** 1 Research Group in Sports Biomechanics (GIBD), Department of Physical Education and Sports, Faculty of Physical Activity and Sport Sciences, Universitat de València, 46010 Valencia, Spain; j.ignacio.priego@uv.es; 2 Research Group in Sports Technique and Tactics (GITTE), Department of Physical Education and Sports, Faculty of Physical Activity and Sport Sciences, Universitat de València, 46010 Valencia, Spain; jose.f.guzman@uv.es; 3 Biovetmed & Sportsci Research Group, Department of Physical Activity and Sport, Faculty of Sport Sciences, University of Murcia, 30720 San Javier, Spain; josepinoortega@um.es; 4 SPORT Research Group (CTS-1024), CIBIS (Centro de Investigación para el Bienestar y la Inclusión Social) Research Center, University of Almería, 04120 Almería, Spain; galcaraz@ual.es

**Keywords:** sand sports, microtechnology, accelerometer, automatic detection, player load

## Abstract

Sports performance initiation is of significant interest in sports sciences, particularly in beach volleyball (BV), where players usually combine indoor and BV disciplines in the formative stages. This research aimed to apply an electronic performance tracking system to quantify the physical-conditional performance of young male BV players during competition, considering age group (U15 or U19), sport specialisation (indoor or beach) and the set outcome (winner or loser). Thirty-two young male players, categorised by age and sport specialisation, were analysed during 40 matches using electronic performance tracking systems (Wimu PRO^TM^). Data collected were the set duration, total and relative distances covered, and number and maximum values in acceleration and deceleration actions. U19 players and BV specialists, compared to their younger and indoor counterparts, covered more distance (719.25 m/set vs. 597.85 m/set; 719.25 m/set vs. 613.15 m/set) and exhibited higher intensity in terms of maximum values in acceleration (4.09 m/s^2^ vs. 3.45 m/s^2^; 3.99 m/s^2^ vs. 3.65 m/s^2^) and deceleration (−5.05 m/s^2^ vs. −4.41 m/s^2^). More accelerations (557.50 n/set vs. 584.50 n/set) and decelerations (561.50 n/set vs. 589.00 n/set) were found in indoor players. Additionally, no significant differences were found in variables regarding the set outcome. These findings suggest that both age and specialisation play crucial roles in determining a great physical-conditional performance in young players, displaying a higher volume and intensity in external load metrics, whereas indoor players seem to need more accelerations and decelerations in a BV adaptation context. These insights highlight the age development and sport specialisation in young volleyball and BV athletes.

## 1. Introduction

Beach volleyball (BV) is an outdoor split-court sport played by pairs on the sand, where environmental and contextual variables influence game performance [1,2]. Its popularity has increased recently, prompting sports science researchers to deepen BV analysis from different perspectives [3]. Technical-tactical [4,5], psychological [6], physiological [2], environmental [7] and reglementary [8] aspects of the game have been studied so far. However, physical-conditional parameters have proved to be one of the most studied aspects of the game, from the beginning years [9,10,11] to the present [12].

Regarding the physical-conditional analysis, BV is an intermittent sport that combines short maximal efforts with prolonged low-intensity recovery periods [2]. These maximum efforts, composed of jumps, defences and displacements with direction changes at high intensity on an irregular and unstable surface such as the sand, make BV a very physically demanding sport [2]. Therefore, players’ internal and external loads during competition and training have been studied extensively for performance monitoring, injury prevention, and health control [1,3,13,14].

When discussing physical-conditional aspects of the game, it is necessary to define the concept of load as the intensity, volume and frequency of stimulus experienced by athletes [15,16]. One of BV’s most studied external load variables has been the number of jumps [17,18]. Still, new research trends indicate that kinematic analysis of the game could better integrate the external workloads by considering variables such as total distances, number and intensity of accelerations, decelerations or changes in direction by the players during the game [1,3,19,20].

Moreover, physical-conditional variables have been shown to differ in terms of age groups [1,21], game outcomes [3,22] and players’ specialisation [3,14,17,21,23]. In terms of age group, a tendency towards longer sets, more jumps and greater distances covered at higher intensities has been found in older categories [1,21]. Concerning game outcomes, winning teams show higher speeds and jumps in blockers and higher adjusting ability, speed, accelerations and decelerations in defenders [3]. As far as players’ specialisation is concerned, different approaches can be proposed. Some authors have used the player’s team role to differentiate between blockers, defenders and universal players [3,14,17,21,23]. Still, to date, no BV articles have considered the players’ specialisation in terms of exclusivity in the BV practice in the formative stages. In this sense, two methodological approaches can be identified: specificity and multidisciplinary [24,25]. Young BV players usually combine indoor and BV seasons in their formative stages. Therefore, two levels of players’ specialisation can be determined: players who train indoors and move to BV during the summer season (indoor) or players with a more specialised dedication to BV (beach). 

For a better understanding of physical game demands, in the last few years, research studies using electronic performance tracking systems (EPTSs) in BV have emerged [1,3,13,14,16], and this trend can be considered as a starting point in the application of this technology in BV. To the best of the authors’ knowledge, these publications have focused on monitoring female BV players, including a case study analysing 99 matches of an elite team [14]; a comparative study (U23 vs. Senior) analysing ten teams at the Australian BV National Championships [1]; two studies focusing on the validation and description of the physical-conditional demands of NCAA players [13,16]; and a study focusing on how contextual variables (player profile, set type, and match outcome) affect data collected with Global Position Systems in six teams during 30 official matches of the Portuguese BV National Championship [3]. Therefore, a gap in the research literature that provides performance indicators obtained with an EPTS in men’s BV is recognised, as well as information related to the initial stages.

For all the above reasons, this research aimed to apply EPTS technology to objectively quantify the physical-conditional performance of young male BV players during competition, considering the age group (U15 vs. U19), the players’ sport specialisation (indoor vs. beach), and the set outcome (winner vs. loser). The following hypotheses are established: (1) volume and intensity external load variables will increase with the players’ age; (2) adjustment variables, such as the number of accelerations and decelerations, will be higher in indoor players; (3) volume and intensity values will be higher in BV specialist players; and (4) intensity external load variables will be higher in the winners’ teams.

## 2. Materials and Methods

### 2.1. Participants

Thirty-two youth men players were analysed according to their age category (U15 or U19) and sport specialisation (indoor—I, or beach—B): (a) U15-I (n = 8, 12 ± 1 years, 43.7 ± 9.1 kg and 1.55 ± 0.11 m, national league level), (b) U15-B (n = 8, 13 ± 1 years, 57.8 ± 10.7 kg and 1.70 ± 0.10 m, national league level), (c) U19-I (n = 8, 14 ± 1 years, 58.1 ± 6.5 kg and 1.76 ± 0.05 m, national league level), and (d) U19-B (n = 8, 18 ± 1 years, 68.1 ± 8.0 kg and 1.79 ± 0.07 m, national league level). All participants were free from injury during testing and voluntarily participated in the study. Players over 18 years old and legal representatives of minors signed an informed consent form giving their assent to participate. The study was conducted according to the guidelines of the Declaration of Helsinki (2013) and approved by the Ethics Committee of the University of Valencia (ID: 2158717).

### 2.2. Procedure

**Figure 1:**
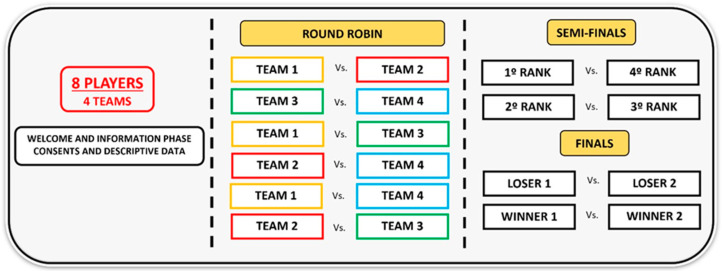
Competition format representation.

Four simulated competitions were organised, one for each group (U15-I, U15-B, U19-I, U19-B), where the 8 players (4 teams) played a total of 10 one-set matches to 21 points, distributed in three phases: round robin, semi-finals and finals (Figure 1). Athletes were briefed on the measurement protocol and the competition system upon arrival. Before each match, the players were instrumented with electronic tracking devices. The starting and finish set time was recorded, and the set outcome was registered, assigning the winning (W) or losing (L) category to the teams based on the game result. A total of 40 four-player matches (n = 160 records) were monitored (20 U15-B records had to be excluded due to technical issues), in which 1310 points (369 in U15-I, 193 in U15-B, 376 in U19-I, 372 in U19-B) and 536 min (166 in U15-I, 74 in U15-B, 133 in U19-I, 163 in U19-B) were played.

### 2.3. Technology

**Figure 2:**
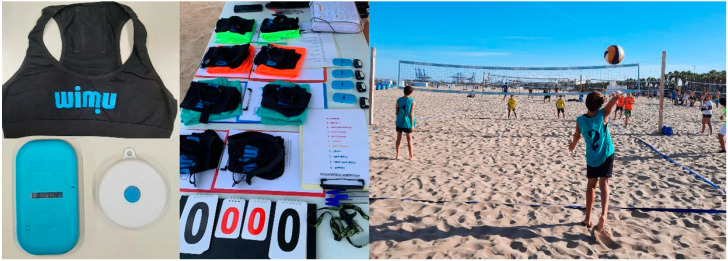
Equipment used during competition monitoring.

WIMU PRO^TM^ (RealTrack Systems, Almeria, Spain), a multi-sensor device with four triaxial accelerometers, three triaxial gyroscopes, a triaxial manometer and a Global Position System, was the EPTS used to monitor players’ physical-conditional performance during competition [26]. This device was validated previously by different sports scenarios [27,28]. Before each match, players wore a tough-fit top with an interscapular (vertebral T2-T4 level) compartment where the WIMU PRO^TM^ device was located [26]. A push button provided by the manufacturer connected via ANT+ technology to the device was used for more precise signal segmentation (start and end of the set) (Figure 2). Data generated by these sensors during the sets were downloaded and processed in the SPRO program (Software version number: Version 1.0.0 Copilation: 989; RealTrack Systems, Almeria, Spain), where the INTERVAL PRO monitor (Software version number: Version 1.0.0 Copilation: 989; RealTrack Systems, Almeria, Spain) was applied to calculate the variables through algorithms configured by the manufacturer.

### 2.4. Measurement

To compare the players’ physical-conditional efforts, nine external load variables were considered to represent the volume and intensity performance: set duration (min), distance covered per set (m/set), distance covered per minute (m/min), number of accelerations and decelerations per set, per minute, and maximum acceleration and deceleration (m/s^2^). Variables were selected considering the results of previous studies [1,3,13,14,29].

### 2.5. Statistical Analysis

A descriptive analysis was carried out in two parts: (i) with the age group (U15 vs. U19), the players’ sport specialisation (indoor vs. beach) and the set outcome (winner vs. loser) separately, and (ii) with the interaction between age group and players’ sport specialisation (U15-I, U15-B, U19-I, U19-B), and age group and set outcome (U15-L, U15-W, U19-L, U19-W). The median (µ) and interquartile range (IQR) were used for data descriptive representation. The Shapiro–Wilk test showed a non-normal data distribution (*p* < 0.05). Therefore, Mann–Whitney U and Kruskal–Wallis tests were applied (*p* < 0.05), as well as the Holm adjustment for pairwise comparison (*p* < 0.05). Furthermore, the effect size (ES) and the confidence interval (CI) were calculated. Rank-biserial correlation (rbis) effect size considering 0.10 small, 0.30 medium and 0.50 large was used for the Mann–Whitney U test [30]. For the Kruskal–Wallis test, the rank epsilon square (*ε*^2^) effect sizes were chosen with values as follows: 0.00–0.01, negligible; 0.01–0.04, weak; 0.04–0.16, moderate; 0.16–0.36, relatively strong; 0.36–0.64, strong; and 0.64–1.00, very strong [31]. RStudio (version 2023.06.0, package “ggstatplot”) software was used in the analysis.

## 3. Results

**Figure 3:**
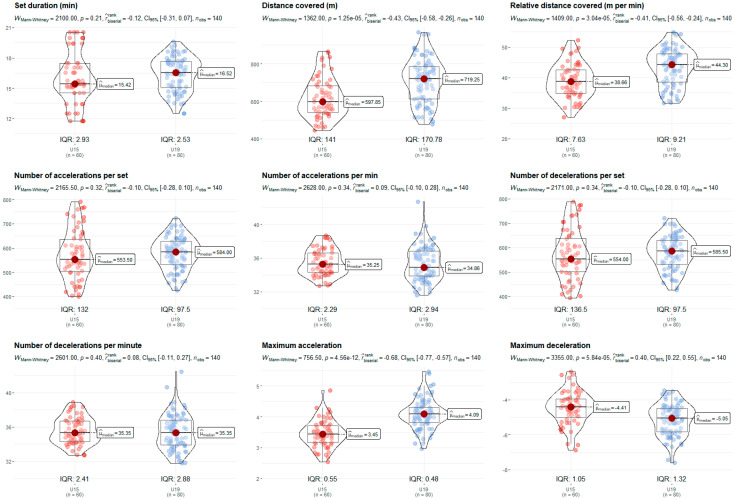
Age group comparison (U15 vs. U19) of the nine performance variables portrayed as violin plots. Median values (µ), interquartile ranges (IQR), Mann–Whitney U test (*p* < 0.05), rank-biserial correlation effect size (rbis), 95% confidence interval (CI_95%_), and number of observations (n_obs_) expressed in each plot.

**Figure 4:**
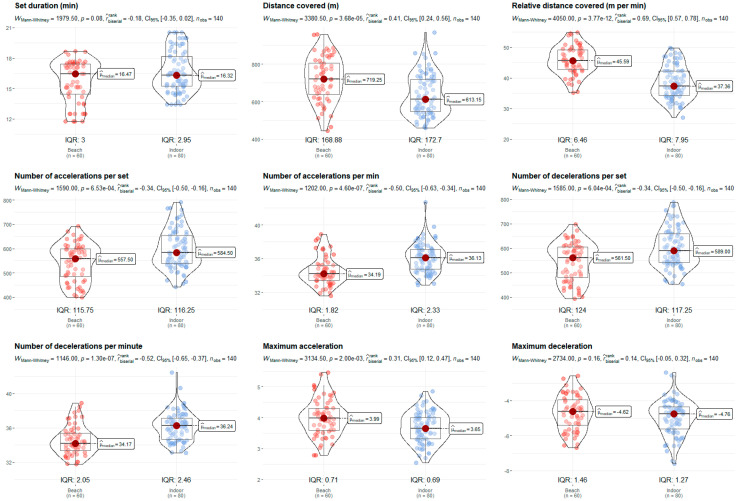
Players’ specialisation comparison (beach vs. indoor) of the nine performance variables portrayed as violin plots. Median values (µ), interquartile ranges (IQR), Mann–Whitney U test (*p* < 0.05), rank-biserial correlation effect size (rbis), 95% confidence interval (CI_95%_), and number of observations (n_obs_) expressed in each plot.

**Figure 5:**
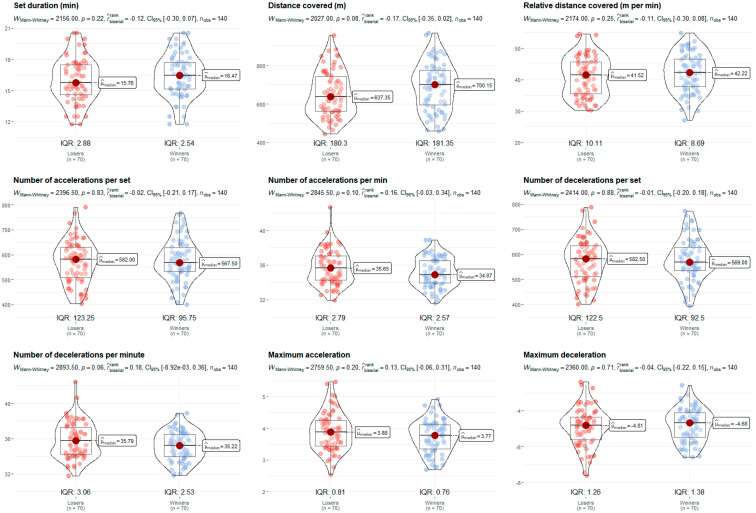
Set outcome comparison (loser vs. winner) of the nine performance variables portrayed as violin plots. Median values (µ), interquartile ranges (IQR), Mann–Whitney U test (*p* < 0.05), rank-biserial correlation effect size (rbis), 95% confidence interval (CI_95%_), and number of observations (n_obs_) expressed in each plot.

The U19 category displayed higher volume and intensity values compared to the U15. The older players covered more distance (719.25 m/set vs. 597.85 m/set, *p* = 0.001, rbis = 0.45) and more relative distance (44.30 m/min vs. 38.66 m/min, *p* = 0.001, rbis = 0.41), and exhibited higher intensity in maximum accelerations (4.09 m/s^2^ vs. 3.45 m/s^2^, *p* = 0.001, rbis = 0.68) and decelerations (−5.05 m/s^2^ vs. −4.41 m/s^2^, *p* = 0.001, rbis = 0.40) (Figure 3). Although the variables of duration and the number of accelerations and decelerations per set and minute did not reveal significant differences, there was a trend towards higher values in older age groups. In relation to Figure 4, data show that BV specialists covered a greater distance (719.25 m/set vs. 613.15 m/set, *p* = 0.001, rbis = 0.41) and relative distance (45.59 m/min vs. 37.36 m/min, *p* = 0.001, rbis = 0.69), but performed fewer accelerations (557.50 n/set vs. 584.50 n/set, *p* = 0.001, rbis = 0.34; 34.19 n/min vs. 36.13 n/min, *p* = 0.001, rbis = 0.50) and decelerations (561.50 n/set vs. 589.00 n/set, *p* = 0.001, rbis = 0.34; 34.17 n/min vs. 36.24 n/min, *p* = 0.001, rbis = 0.52) per set and minute. However, they did perform higher maximum accelerations (3.99 m/s^2^ vs. 3.65 m/s^2^, *p* = 0.001, rbis = 0.31). Moreover, no differences were found considering the set outcome (Figure 5).

In the category-level interaction (Table 1), the U19-B group covered the greatest distances (760.90 m/set, *p* = 0.001, *ε*^2^ = 0.23). A tendency to cover more distance with more age and BV specificity was shown. Similar trends were found in relative distance but with higher values in U15-B and U19-B, regardless of age. In terms of accelerations and decelerations (both in volume and intensity), the lower-specialisation groups tended to have higher values. Significant differences were found between the U19-I and the U15-B and U19-B groups, but no differences between U15-I and U19-I were shown. Additionally, U15-I had higher values in decelerations/set compared to U15-B, and U15-I had higher values in the intensity variables (accelerations/min and decelerations/min) compared to U19-B. Maximum values of accelerations and decelerations tended to be higher in older categories, with the higher acceleration values in U19-I.

Finally, in the category–result interaction (Table 2), the older age groups’ tendency to present higher values in total distance, relative distance, maximum acceleration, and deceleration was confirmed. Moreover, no significant differences were found between the winning and losing teams within each category (U15 vs. U19), confirming the results obtained in Figure 3.

## 4. Discussion

This study aimed to determine how the physical-conditional variables evolve in men’s BV competition, considering age group (U15 vs. U19), players’ sport specialisation (indoor vs. beach), and the set outcome (winner vs. loser). It is important to consider that our study aimed to enhance ecological validity by analysing performance variables in real-game contexts and ensuring the findings represent actual competition dynamics. This approach, however, may impact internal validity, as factors like years of training and physical development were not explicitly controlled. While such variables could influence the outcomes, our primary objective was a descriptive performance comparison across levels and categories, prioritising real-world applicability over strict control of confounding factors. The hypotheses proposed by the authors were as follows: (1) volume and intensity of external load variables would increase with players’ age, and (3) volume and intensity values would be higher in beach specialist players, both of which were accepted; (2) adjustment variables, such as the number of accelerations and decelerations, would be higher in indoor players, which was partially accepted; and (4) the intensity external load variables would be higher in winning teams, which was rejected.

To start with, older players can sustain higher-intensity efforts for extended periods, achieving greater values in both total and relative distances covered with higher-intensity accelerations and decelerations. These findings align with the existing literature, which has shown that senior players cover greater relative distance, at higher speeds, with more rest periods, and perform more jumps compared to the younger category [1,21]. This suggests an increase in performance metrics as players grow, regardless of the level of specialisation. In this sense, the higher values are attributed to an increase in physical and metabolic capacity as a result of maturation and growth, as well as more experience, shown in better decision-making and skills control [32].

Furthermore, volume and intensity values were higher in BV specialists, as they covered greater distances and relative distances compared to indoor players, as shown in previous studies [12]. Despite performing fewer accelerations and decelerations per set and minute, beach specialist players exhibited higher maximum accelerations. These suggest better specificity adaptation to the BV requirements, playing at a higher intensity without needing extra accelerations and decelerations for adjustments. In this sense, indoor players need to adapt to the open context of the sand surface, wind and sun, as well as to the larger player responsibility area, making it difficult to maintain beach specialist external load volume and intensity [12].

Regarding accelerations and decelerations, indoor players are used to specific position roles, having to adapt in BV to a general role using all volleyball skills (serve, receive, set, spike, block and defend), and needing an additional number of accelerations and decelerations for in-game adjustments [12]. Specifically, the study found that indoor players tend to have slightly higher values in decelerations/set compared to BV specialists in the U15 category and higher accelerations/min and decelerations/min compared to U19 beach specialist players. These findings suggest that while indoor players may perform more frequent adjustments, the differences are not pronounced enough to be statistically significant in all categories.

Finally, no significant differences were found between winning and losing teams regarding the volume and intensity of external load variables within each age category. This finding is consistent across the metrics of total distance, relative distance, maximum acceleration, and deceleration. It provides differing results from recent reference studies on elite senior female athletes, where winning teams show higher speeds and jumping in blockers, and higher adjusting ability, speed, accelerations and decelerations in defenders [3]. This is probably because, in senior elite categories, the physical-conditional aspect of the game becomes more determined than in formative stages. These results suggest that while physical performance is crucial, other factors such as technical skill, tactical execution, and psychological aspects of the game may play more critical roles in determining the outcome of the matches in formative categories.

One major limitation of this study is determining the effect of the number of sets on performance indicators, as the data were collected from one-set simulated competitions. This setup may not accurately reflect the typical accumulative load and fatigue experienced in multi-set matches, although many U15 and U19 competitions present one-set match formats. Moreover, recruiting top elite athletes at this formative stage also becomes a significant challenge, which might have impacted the sample’s representativeness.

Moreover, the study was conducted on different days, making it difficult to control contextual variables such as wind, heat, and humidity, which can significantly influence performance. This variability adds a layer of complexity when interpreting the results, as these environmental factors can affect players’ physical and technical performances. Furthermore, the study did not control other contextual variables, such as technical, tactical, or psychological aspects, which can also impact performance. These uncontrolled factors may confound the results, making it challenging to isolate the effects of the measured performance indicators. Future research should aim to include a larger, more diverse sample and consider multi-set matches to provide a more comprehensive understanding of performance indicators in men’s BV at the formative stages. Additionally, these protocols should be implemented for female formative and professional players to assess potential gender differences, providing valuable insights into performance variations and promoting a more inclusive understanding of the sport. Furthermore, studies should also explore the progression of athletic and technical performance across a broader range of age groups and training levels, as this could clarify how longer training years and competitive exposure influence development in beach volleyball athletes.

## 5. Conclusions

The findings indicate that age and sports specialisation significantly influence the physical-conditional performance of young volleyball players measured with EPTS technology. Older players and those specialised in BV show higher volume and intensity in their external load metrics, whereas indoor players perform more acceleration and deceleration movements. Additionally, the set outcome does not significantly impact the external load variables.

The findings of this study offer valuable insights for coaches and trainers in developing training programs for young volleyball players. The significant influence of age and sports specialisation on physical-conditional performance suggests that training volume and intensity should be consciously adapted to these factors. Older players and those specialised in BV demonstrate higher volumes and intensities in their physical activities. Therefore, training programs for these athletes should include higher intensity and volume exercises to match their advanced physical capabilities.

Moreover, the finding related to set outcomes highlights the need for coaches to consider other factors, such as technical skills, tactical decisions and psychological aspects of the game, when preparing athletes for competition in the formative stages. Furthermore, by integrating EPTS technology into regular training and match analysis, coaches can establish reference values for different age groups and specialisations, enhancing their ability to monitor and adjust training loads effectively. This approach not only helps optimise performance but also prevents injuries by ensuring that young athletes are not overtraining.

These data and differences found between indoor and BV may be suitable for a proper fusion of experiences in both sports, ensuring a wider physical stimulus favouring future sports specialisation and injury prevention.

## Figures and Tables

**Table 1:** Comparative interaction between age group and players’ specialization.

Variables	U15-I µ (IQR)	U15-B µ (IQR)	U19-I µ (IQR)	U19-B µ (IQR)	X^2^	p	*ε* ^2^	CI_95%_	Holm					
Set duration (min)	15.59 (4.20)	15.12 (4.57)	16.42 (2.79)	16.60 (2.44)	7.68	0.05	0.06	[0.01, 1.00]				†		
Distance covered (m/set)	585.95 (129.60)	643.50 (163.45)	682.15 (185.30)	760.90 (163.28)	32.25	0.01	0.23	[0.16, 1.00]			⁂		‡	#
Relative distance covered (m/min)	35.72 (6.11)	43.95 (7.20)	39.55 (10.31)	46.48 (5.77)	57.71	0.01	0.41	[0.34, 1.00]	*	⁑	⁂			#
Accelerations per set (n/set)	565.50 (138.75)	506.00 (168.00)	605.00 (84.00)	571.00 (105.25)	14.16	0.01	0.10	[0.04, 1.00]				†		#
Accelerations per min (n/min)	35.72 (2.29)	34.69 (1.84)	36.39 (2.06)	33.97 (1.65)	26.99	0.01	0.21	[0.13, 1.00]			⁂	†		#
Decelerations per set (n/set)	567.50 (139.25)	498.50 (165.25)	606.50 (88.50)	570.50 (104.75)	14.32	0.01	0.10	[0.05, 1.00]	*			†		#
Decelerations per min (n/min)	35.94 (2.17)	34.88 (2.03)	36.56 (2.06)	34.13 (2.10)	30.46	0.01	0.22	[0.14, 1.00]			⁂	†		#
Maximum acc. (m/s^2^)	3.45 (0.51)	3.42 (0.49)	3.99 (0.58)	4.16 (0.66)	53.37	0.01	0.38	[0.29, 1.00]		⁑	⁂	†	‡	#
Maximum dece. (m/s^2^)	−4.65 (1.23)	−3.79 (0.88)	−5.09 (1.42)	−4.99 (1.28)	26.63	0.01	0.19	[0.14, 1.00]	*			†	‡	

(U15-I) Under 15 age group indoor specialisation, (U15-B) under 15 age group beach specialisation, (U19-I) under 19 age group indoor specialisation, (U19-B) under 19 age group beach specialisation. (µ) Median values, (IQR) interquartile ranges, (X^2^) Kruskal–Wallis (*p* < 0.05), (*ε*^2^) Rank Epsilon Square Effect Sizes, (CI_95%_) 95% Confidence Interval. Holm adjustment (*p* < 0.05) pairwise differences is represented as: (*) U15-I vs. U15-B, (⁑) U15-I vs. U19-I, (⁂) U15-I vs. U19-B, (†) U15-B vs. U19-I, (‡) U15-B vs. U19-B, (#) U19-I vs. U19-B.

**Table 2:** Comparative interaction between age group and set outcome.

Variables	U15-I µ (IQR)	U15-B µ (IQR)	U19-I µ (IQR)	U19-B µ (IQR)	X^2^	p	*ε* ^2^	CI_95%_	Holm					
Set duration (min)	15.42 (2.96)	15.42 (2.69)	16.34 (2.83)	16.34 (2.19)	4.23	0.24	0.03	[0.01, 1.00]						
Distance covered (m/set)	588.60 (115.58)	605.05 (163.76)	687.80 (199.42)	734.70 (109.00)	22.86	0.01	0.16	[0.08, 1.00]		⁑	⁂		‡	
Relative distance covered (m/min)	37.70 (7.83)	39.23 (8.51)	43.81 (10.52)	44.86 (8.12)	18.74	0.01	0.13	[0.07, 1.00]		⁑	⁂		‡	
Accelerations per set (n/set)	568.50 (140.75)	541.50 (100.50)	585.00 (114.50)	581.00 (88.75)	1.84	0.61	0.01	[0.01, 1.00]						
Accelerations per min (n/min)	35.73 (2.48)	34.92 (2.52)	35.32 (3.00)	34.84 (3.02)	3.67	0.30	0.03	[0.01, 1.00]						
Decelerations per set (n/set)	572.00 (140.25)	545.00 (104.50)	586.50 (118.25)	585.00 (86.75)	1.72	0.63	0.01	[0.01, 1.00]						
Decelerations per min (n/min)	35.71 (2.42)	35.19 (2.38)	35.79 (3.25)	35.22 (2.69)	4.15	0.25	0.03	[0.01, 1.00]						
Maximum acc. (m/s^2^)	3.52 (0.42)	3.30 (0.56)	4.14 (0.63)	4.02 (0.42)	50.54	0.01	0.36	[0.28, 1.00]		⁑	⁂	†	‡	
Maximum dece. (m/s^2^)	−4.38 (0.84)	−4.53 (1.41)	−5.14 (1.26)	−4.95 (1.42)	18.05	0.01	0.13	[0.07, 1.00]		⁑	⁂	†		

(U15-L) Under 15 age group loser team, (U15-W) under 15 age group winner team, (U19-L) under 19 age group loser team, (U19-B) under 19 age group winner team. (µ) Median values, (IQR) Interquartile ranges, (X^2^) Kruskal–Wallis (*p* < 0.05), (*ε*^2^) Rank Epsilon Square Effect Sizes, (CI_95%_) 95% Confidence Interval. Holm adjustment (*p* < 0.05) pairwise differences is represented as: (⁑) U15-L vs. U19-L, (⁂) U15-L vs. U19-W, (†) U15-W vs. U19-L, (‡) U15-W vs. U19-W.

## Data Availability

Data are contained within the article.
